# Carcinosarcoma in Ovarian Mature Teratoma: A Rare Autopsy Case

**DOI:** 10.7759/cureus.87629

**Published:** 2025-07-09

**Authors:** Nobuyasu Ikai, Hidetaka Sato, Yoko Yamada, Suzuko Moritani, Hiyo Obikane

**Affiliations:** 1 Department of Diagnostic Pathology, Tokyo Metropolitan Police Hospital, Tokyo, JPN; 2 Department of Obstetrics and Gynecology, Tokyo Metropolitan Police Hospital, Tokyo, JPN; 3 Department of Diagnostic Pathology, Shiga University of Medical Science, Otsu, JPN

**Keywords:** autopsy, carcinosarcoma, ovary, sarcoma, squamous cell carcinoma, teratoma

## Abstract

Malignant transformation of an ovarian mature teratoma is a rare phenomenon. The most common malignant tumor is squamous cell carcinoma, followed by other histologic subtypes. Carcinosarcoma in ovarian mature teratoma is much less common, with only several case reports in the literature, and their autopsies have been rarely reported. Here we report a rare autopsy-documented case of ovarian mature teratoma, which has both squamous cell carcinoma and sarcomatous components. The right adnexectomy specimen of a 45-year-old female patient contained non-invasive (in situ) and invasive squamous cell carcinoma and spindle cell sarcoma, which were juxtaposed with transitions between the two, epithelial (carcinomatous) and mesenchymal (sarcomatous), components. The patient died seven weeks after surgery, and an autopsy was performed, revealing significant peritoneal dissemination. For the intraperitoneal dissemination, all were histologically spindle cell sarcoma components, and no squamous cell carcinoma components were identified, suggesting that sarcomatous overgrowth occurred. Sarcomatous components also metastasized to the lung. Death was attributed to peritoneal dissemination of the sarcomatous component. In this case, extensive tissue sampling was performed both in the adnexectomy specimen and the autopsy specimen to conclude that sarcomatous overgrowth in carcinosarcoma is the cause of death.

## Introduction

Malignant transformation of a mature ovarian teratoma is a rare phenomenon, predominantly in the postmenopausal period; less than 2% of teratomas undergo such a malignant transformation [1,2]. The most common malignant tumor occurring in ovarian mature teratomas is squamous cell carcinoma, followed by adenocarcinoma and other histologic subtypes, including malignant melanomas, sebaceous gland carcinomas, small cell carcinomas, basal cell carcinomas, transitional cell carcinomas, undifferentiated carcinomas, sarcomas, and lymphoma [1].

Carcinosarcoma, defined as a malignant epithelial neoplasm exhibiting mesenchymal differentiation, resulting in a biphasic morphology, is much less common in ovarian mature teratoma, with only a few case reports in the literature. Among reports of carcinosarcoma arising in mature teratomas, the most common carcinomatous component is squamous cell carcinoma [3-9], followed by adenocarcinoma [9,10]. Some cases have been treated with postoperative chemotherapy, but they often have a poor prognosis. Only a minority of patients survived without recurrence, whereas the majority succumbed to the tumor. Although some fatal cases have been reported, autopsies have been rarely performed. Therefore, whether the epithelial (carcinomatous) or mesenchymal (sarcomatous) components of carcinosarcoma caused the lethal systemic dissemination and metastasis remains unclear. Understanding which component drives dissemination and metastasis is crucial for prognostication and management, but this distinction has rarely been confirmed via autopsy.

To improve the clinicians’ awareness of the tumor, here we report a rare autopsy-documented case of a 45-year-old female patient with ovarian teratoma, which has both squamous cell carcinoma and sarcomatous components, and also describe her rapidly progressive clinical course.

## Case presentation

**Figure 1:**
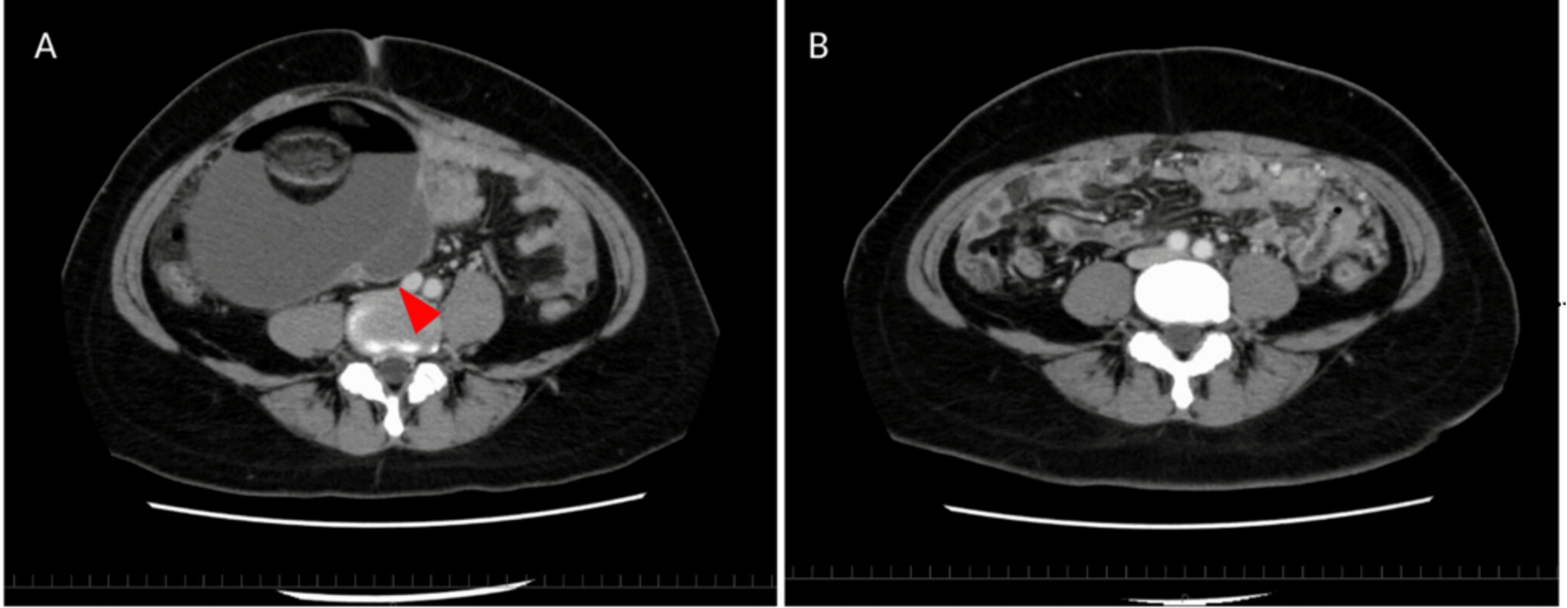
Computed tomography shows peritoneal dissemination and ascites (A) Preoperative CT reveals an ovarian mature teratoma  (red arrowhead) with no evidence of peritoneal dissemination or ascites. (B) Postoperative CT showing peritoneal dissemination and ascites.

A 45-year-old female patient’s right ovarian mature teratoma was untreated for approximately 20 years. The patient had no significant past medical history apart from the teratoma. Eight weeks before her death, she developed intermittent pain in the upper right abdomen, and then it spread to the lower abdomen. Preoperative computed tomography (CT) revealed torsion of the 16 cm teratoma pedicle without peritoneal dissemination or ascites (Figure 1A). The following serum tumor markers were elevated: carcinoembryonic antigen (CEA), carbohydrate antigen (CA) 19-9, cancer antigen (CA) 125, and squamous cell carcinoma (SCC) antigen (Table 1). An emergency abdominal right adnexectomy was performed, and she was discharged uneventfully one week after surgery (six weeks before death). Two weeks post-discharge (four weeks before death), she experienced fatigue, and repeat CT revealed peritoneal dissemination with ascites (Figure 1B). Chemotherapy was deemed unfeasible due to a rapid decline in the patient’s physical condition. The patient received only supportive care and subsequently died without chemotherapy; an autopsy was performed.

**Table 1:** Elevated serum tumor markers Patient laboratory evaluation at admission revealed that serum tumor markers were elevated. CEA: carcinoembryonic antigen, CA 19-9: carbohydrate antigen 19–9, CA125: cancer antigen 125, SCC: squamous cell carcinoma antigen.

Parameter	Patient values	Reference range
CEA	70.7	0-5.0 ng/mL
CA19-9	6185	0-37 U/mL
CA125	443.7	0-35 U/mL
SCC	18.3	0-2.5 ng/mL

**Figure 2:**
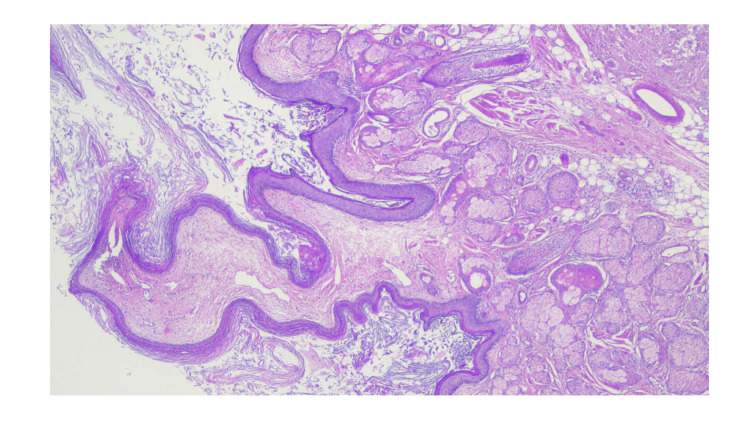
Histopathological features of ovarian mature teratoma Skin tissue on cyst wall of ovarian mature teratoma.

The right adnexectomy specimen grossly contained a 120 mm ovarian cyst filled with hair; a 70 mm segment of its wall was focally thickened to 20 mm. Microscopically, the cyst wall was lined with skin tissue (Figure 2).

**Figure 3:**
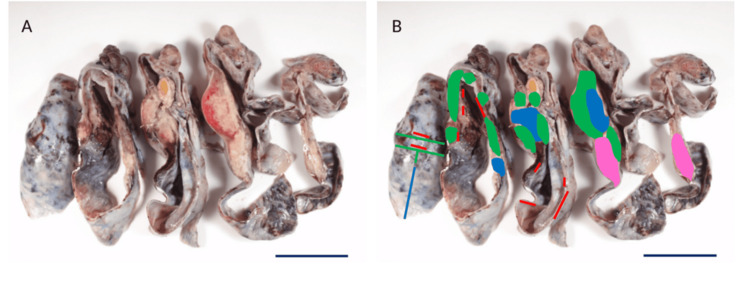
Squamous cell carcinoma and sarcoma juxtaposed in ovarian mature teratoma (A) Cross-section of an ovarian mature teratoma. The cyst wall was thickened to 20 mm. (B) Cross-section with mapping. Red= squamous cell carcinoma in situ, pink= invasive squamous cell carcinoma, green= spindle cell sarcoma, blue= necrosis, black scale bar = 5 cm.

**Figure 4:**
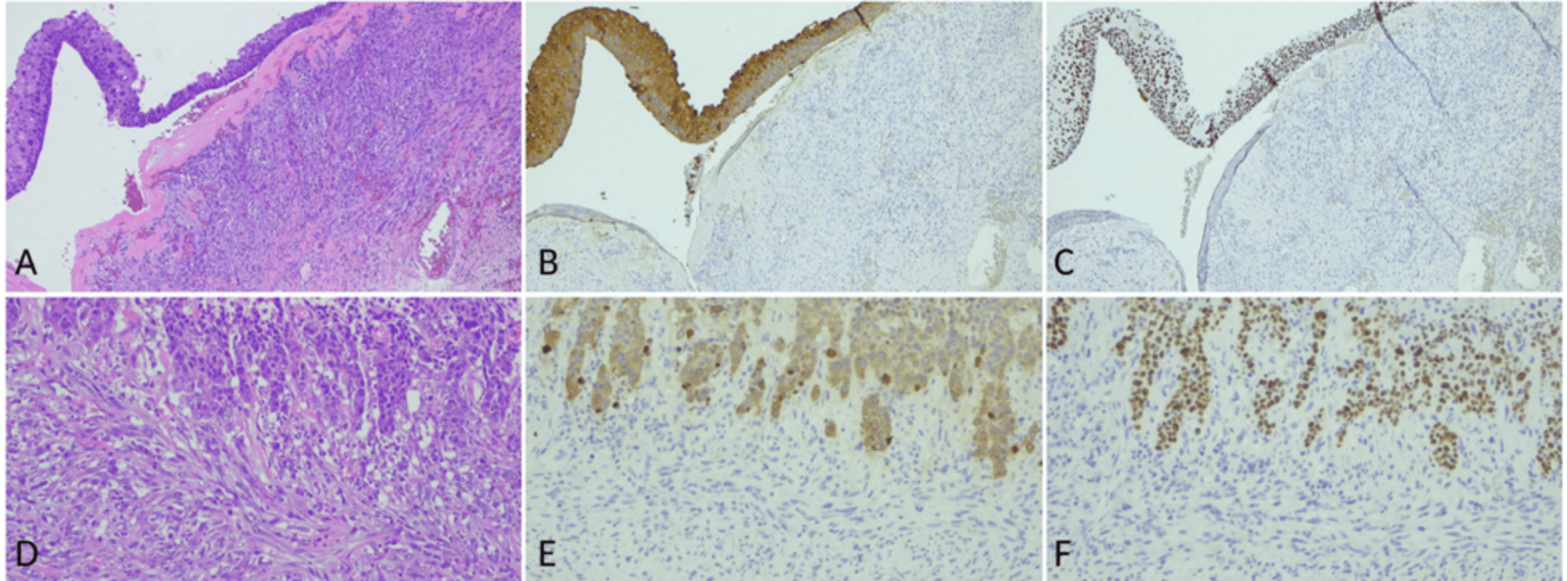
Squamous cell carcinoma with sarcomatous transformation arising in an ovarian mature teratoma (A-C) Squamous cell carcinoma in situ and spindle cell sarcoma. (D-F) Invasive squamous cell carcinoma and spindle cell sarcoma transition. (A, D) Hematoxylin and eosin staining; (B, E) pan-cytokeratin AE1/AE3; and (C, F) p40 staining. Pan-cytokeratin AE1/AE3 and p40 are positive for squamous cell carcinoma, both in situ and invasive, but negative for spindle cell sarcoma.

Within the thickened area, non-invasive (in situ) and invasive squamous cell carcinoma and spindle cell sarcoma were juxtaposed, with transitions between the two, epithelial (carcinomatous) and mesenchymal (sarcomatous) components (Figures 3A, 3B, 4A, 4D). Immunohistochemically, the squamous cells were positive for pan-cytokeratin AE1/AE3, p40, and p63, whereas the sarcomatous cells were negative for all three markers (Figures 4B, 4C, 4E, 4F). Severe venous and lymphatic invasions were observed. Neither immature nor ectopic components were present. Considering the presence of squamous cell carcinoma in situ and transitional zones between carcinoma and sarcoma, the squamous cell carcinoma likely originated within the teratoma and subsequently underwent sarcomatous transformation, which means carcinosarcoma arising in a mature cystic teratoma. The ratio of squamous cell carcinoma to spindle cell sarcoma was approximately 1:5, indicating that most components were sarcomas; however, a small component of squamous cell carcinoma was also observed. During surgery, peritoneal dissemination was not evident, even to the naked eye. The cyst was punctured and aspirated, the adnexa removed, and the peritoneal cavity irrigated with 2000 mL of saline, making intraoperative tumor seeding unlikely. The pathological stage was pT1c1 (TNM) and International Federation of Gynecology and Obstetrics (FIGO) stage IC1.

**Figure 5:**
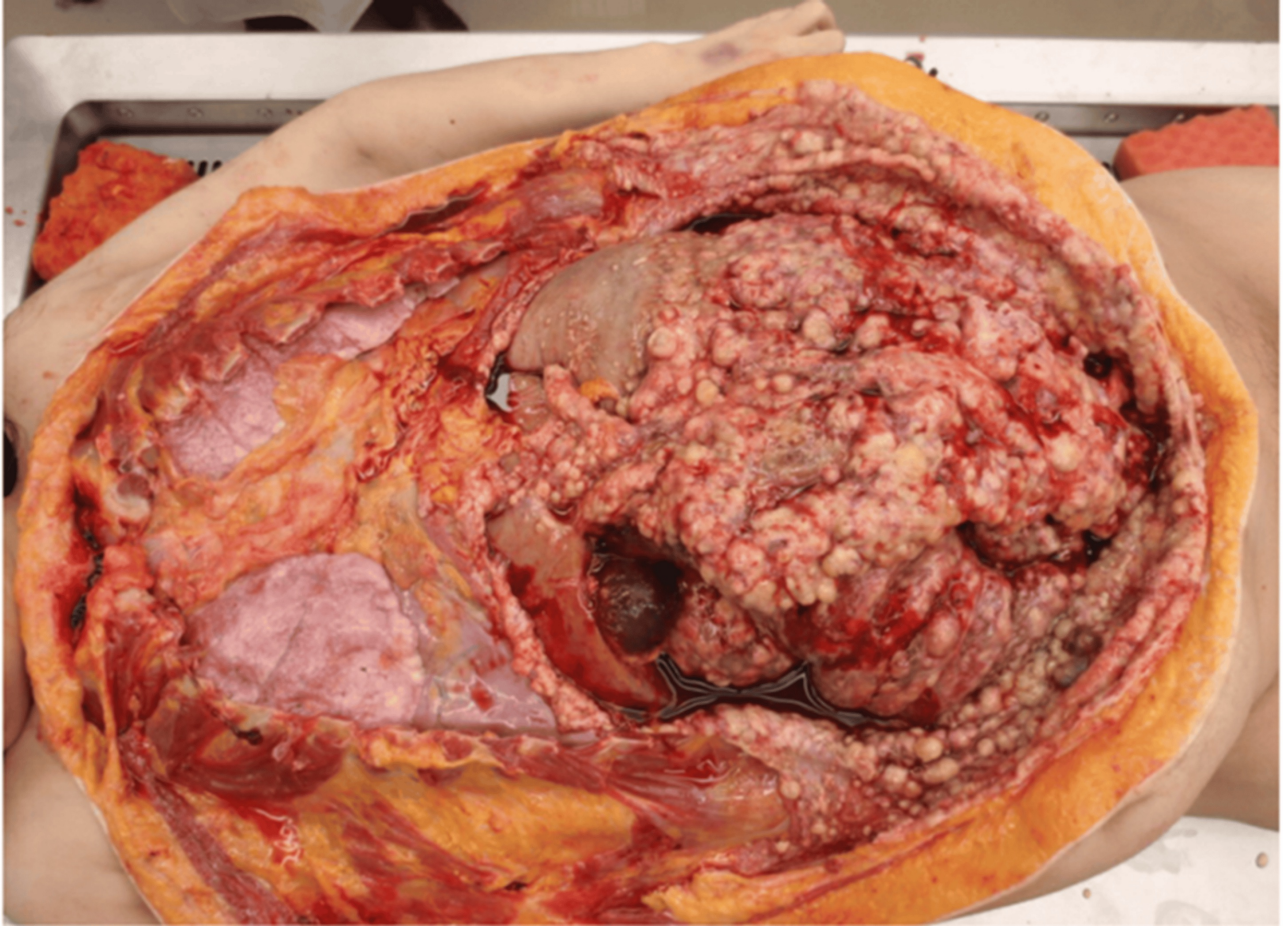
Autopsy reveals significant peritoneal dissemination Autopsy reveals significant peritoneal dissemination (large intestine, small intestine, stomach, gallbladder, liver, uterus, urinary bladder, peritoneum, and omentum) and a large amount of ascites.

**Figure 6:**
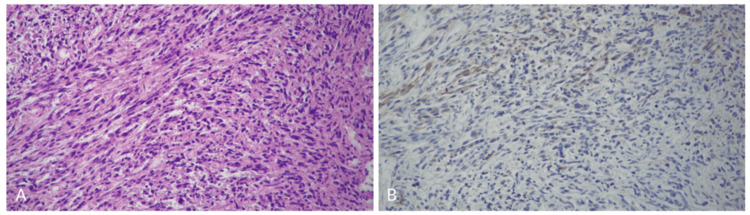
Autopsy specimens are sarcomatous components (A) All autopsy specimens examined are sarcomatous components. (B) Pan-cytokeratin AE1/AE3 immunostaining was weakly positive in only 5% of spindle cell sarcomas.

Autopsy revealed significant peritoneal dissemination (large intestine, small intestine, stomach, gallbladder, liver, uterus, urinary bladder, peritoneum, and omentum) and a large amount of ascites (Figure 5). For the intraperitoneal organs, 36 specimens were randomly collected to examine the histological features of peritoneal dissemination. All were histologically spindle cell sarcoma components, and no squamous cell carcinoma components were identified, suggesting that sarcomatous overgrowth occurred. Immature or ectopic components were not observed. Pan-cytokeratin AE1/AE3 immunostaining was performed on 12 of these specimens; however, approximately 5% of spindle cell sarcomas exhibited only weak positivity, suggesting an epithelial origin but not confirming carcinoma, including squamous cell carcinoma (Figures 6A, 6B). Sarcomatous components also metastasized to the lung, whereas lymph nodes remained free of tumor. Death was attributed to peritoneal dissemination of sarcomatous components and sepsis, with gastrointestinal bleeding secondary to peritoneal involvement as a possible contributing factor.

## Discussion

Fewer than 2% of ovarian mature teratomas undergo malignant transformation, with 80% of these cases being squamous cell carcinomas and 7% being adenocarcinomas [1,2]. Other known cases include malignant melanomas, sebaceous gland carcinomas, small cell carcinomas, basal cell carcinomas, transitional cell carcinomas, undifferentiated carcinomas, sarcomas, and lymphoma [1]. Carcinosarcoma in ovarian mature teratoma is much less common, with only a few case reports in the literature. Among reports of carcinosarcoma arising in mature teratomas, the most common carcinomatous component is squamous cell carcinoma (squamous cell carcinoma with high-grade spindle cell sarcoma [3], moderately-differentiated squamous cell carcinoma with stromal component [4], squamous cell carcinoma and sarcoma [5], squamous cell carcinoma with sarcomatous component of spindle and pleomorphic cells with a high nuclear-cytoplasmic ratio [6], poorly differentiated squamous cell carcinoma and high grade osteosarcoma [7], leiomyosarcoma with a minor component of squamous cell carcinoma [8]), followed by adenocarcinoma (poorly differentiated signet ring cells and plump mesenchymal cells with high nuclear cytoplasmic ratios [10]) and both squamous cell carcinoma and adenocarcinoma with malignant neuroectodermal components [9]. Malignant transformation in ovarian mature teratoma typically occurs in patients over 55 years, with tumors exceeding 100 mm in diameter and elevated tumor markers, such as CEA [1]. Although our patient was younger than 55, her tumor measured 120 mm, and all examined markers (CEA, CA 19-9, CA 125, and SCC antigen) were elevated. Among 188 cases of mature teratomas with malignant transformation to squamous cell carcinoma, the five-year survival rates were 100% for carcinoma in situ, 75.7% for stage I, 38.8% for stage II, 20.6% for stage III, 0% for stage IV, and 48.4% for all stages [2]. Among the 89 patients with stage I disease, 20 died, 18 of whom within two years of diagnosis [2]. Our patient with stage I carcinosarcoma in teratoma progressed rapidly and died seven weeks after surgery.

During the patient’s life, both squamous cell carcinoma and sarcoma were found in the ovary, with sarcoma accounting for a larger proportion (Figure 3B). However, at the time of autopsy, no squamous cell carcinoma was found, with only the sarcoma spreading to the peritoneum and metastasizing to distant sites (Figures 5, 6). These findings suggest that the squamous cell carcinoma originated in a mature teratoma and subsequently underwent sarcomatous transformation, resulting in carcinosarcoma. Subsequently, sarcomatous overgrowth occurs through dissemination and metastasis. Among reports of carcinosarcoma, some cases have been treated with postoperative chemotherapy, including carboplatin with paclitaxel or doxorubicin with cisplatin [3,4], but often have a poor prognosis. Only a minority of patients survived without recurrence, but many died [4,7,8]. Although some fatal cases have been reported, autopsies have been rarely performed. Therefore, whether the epithelial (carcinomatous) or mesenchymal (sarcomatous) components of carcinosarcoma caused the lethal systemic dissemination and metastasis remains unclear. In our case, extensive tissue sampling and mapping were performed both in the adnexectomy specimen and the autopsy specimen to conclude that sarcomatous overgrowth in carcinosarcoma is the cause of death.

Seven weeks post-surgery, the patient’s rapid decline and eventual death suggest sarcomatous transformation as the driving factor. Three hypotheses may explain why peritoneal dissemination was undetected preoperatively and intraoperatively but became evident two weeks later. First, intraoperative seeding may occur, but this is unlikely given the surgical procedure. Second, microscopic peritoneal metastases-undetectable at surgery-were likely already present, as evidenced by significant vascular invasion in the ovarian specimen. Third, her preoperative abdominal pain may have reflected minor cyst rupture in addition to torsion, facilitating tumor spread.

## Conclusions

This report represents a rare autopsy case of carcinosarcoma arising from a mature ovarian teratoma, in which squamous cell carcinoma underwent sarcomatous transformation. The tumor progressed rapidly, and the patient died within weeks after surgery. Autopsy findings revealed that the cause of death was widespread peritoneal dissemination and distant metastasis of the sarcomatous component. This case highlights several important points: Although malignant transformation of mature teratomas is rare, it carries a very poor prognosis when it occurs. In carcinosarcoma, the sarcomatous component can become dominant and drive aggressive systemic spread. Even when no dissemination is observed intraoperatively, microscopic metastases or tumor spread due to cyst rupture may already be present. This case provides valuable pathological evidence of the progression and lethality of carcinosarcoma arising in a mature teratoma. Therefore, in patients with mature teratomas, especially those with high-risk features such as older age, large tumor size, and elevated tumor markers, early surgical intervention and close postoperative monitoring are essential.
